# Empowering Caregivers Through Technology: An Intergenerational Approach to Digital Literacy & Connection

**DOI:** 10.1093/geroni/igaf122.2187

**Published:** 2025-12-31

**Authors:** Cathy Tompkins, Emily Ihara, Skye Leedahl, Jill Juris

**Affiliations:** George Mason University, Fairfax, Virginia, United States; George Mason University, Fairfax, Virginia, United States; University of Rhode Island, Kingston, Rhode Island, United States; Appalachian State University, Boone, North Carolina, United States

## Abstract

Many older adult caregivers struggle to find reliable resources due to limited digital literacy, increasing their stress. Intergenerational digital literacy programs, like the University of Rhode Island’s (URI) Cyber-Seniors, help bridge this gap by enhancing older adults’ tech skills and fostering student connections. This mixed-methods research presentation explores pretest survey findings from a pilot program modeled after URI Cyber-Seniors. Six college students work with nine family caregivers to improve technology skills and access caregiving resources. A pretest survey, using a modified COCOA measurement tool, Geriatrics Attitudes Scale, and Relational Aging Anxiety Scale, assesses perceptions of aging and intergenerational relationships. Open-ended questions explore students’ views on aging, career interests, and older adults’ impressions of younger generations. The program includes five hours of training and runs for five weeks, with weekly student-caregiver meetings. Three students are in an MSW program, two are undergraduate social work majors, and one is a doctoral student. All identify as female, aged 18-29; 33% are white, and 33% multiracial. Caregivers range from 50-89 years old, 78% female, and 55% white. Using grounded theory analysis, emerging student themes range from discomfort working with older adults to strong enthusiasm for intergenerational engagement. Caregivers frequently describe younger generations by referencing technology proficiency but often include negative descriptors such as “challenged,” “unaware,” or “entitled.” This discussion will explore generational perceptions, shared strengths, and motivations for engaging in intergenerational programs.

